# Life cycle inventory of Miscanthus production on a commercial farm in the US

**DOI:** 10.3389/fpls.2023.1029141

**Published:** 2023-07-27

**Authors:** Paul R. Adler

**Affiliations:** United States Department of Agriculture, Agricultural Research Service (USDA-ARS), Pasture Systems and Watershed Management Research Unit, University Park, PA, United States

**Keywords:** bioenergy, biomass, greenhouse gas emissions (GHG emissions), life cycle assessment - LCA, miscanthus, nitrous oxide - N2O, remote sensing - GIS, soil carbon (C) sequestration

## Abstract

There has been considerable interest in use of Miscanthus (Miscanthus x giganteus) as a feedstock for bioenergy production due to its potential to reduce greenhouse gas emissions associated with cellulosic feedstock production and more recently for alternative uses as a biomass crop. To date, data on Miscanthus production in the US has been based on small scale research plots due to the lack of commercial scale production fields. Research plot yields are often much higher than commercial fields for a variety of reasons including reduced spatial variability and location on better quality farmland. The objectives of this study were to quantify the inputs for production of Miscanthus at the commercial farm scale, evaluating methods to characterize fuel use for establishment and management of Miscanthus production and using satellite data to characterize spatial yield variation of production fields. We logged energy use on agricultural machinery from Miscanthus production planted on more than 1000 ha of land and modeled N_2_O emissions and changes in soil carbon using DayCent. Although fuel use was higher for land preparation in fields with perennial vegetation, fuel to harvest Miscanthus dominated greenhouse gas (GHG) emissions (>90%) from agriculture machinery for crop management. The N_2_O emissions and changes in soil carbon were the largest source and sink of GHG emissions associated with Miscanthus production, respectively. Although ~ 50% of the established lands had Miscanthus yields < 5 Mg/ha, yields needed to be > 5 Mg/ha for ΔSOC to be positive. Given the large impact of yield on ΔSOC, net GHG for Miscanthus production with yields of 5 to 25 Mg/ha ranged ~130 to -260 kg CO_2_e/Mg biomass. Use of both energy use for Miscanthus harvest and satellite imagery were good methods to characterize spatial variability of commercial production fields. This demonstrates the potential to use this within field yield data to better understand factors driving subfield yield variability and use of satellite data to quantify early yield predictions.

## Introduction

Biomass crops such as warm-season grasses are being used for feed, fiber, and fuel. Although commercial scale production of cellulosic ethanol has been slow to develop, other commercial scale uses of dedicated biomass crops such as Miscanthus (Miscanthus x giganteus) have developed, such as a source of fiber for compostable packaging, combustion, animal bedding, erosion control, as an absorbent, and fiber supplement for pet food (http://mfiber.net; https://aggrowtech.com/biomass/). Marginal and abandoned or idle farmlands could be used for perennial biomass crop production, providing both economic and environmental benefits (Field et al., 2008; Richards et al., 2014; Khanna et al., 2021). Lands enrolled in the Conservation Reserve Program (CRP) could meet this goal while maintaining the environmental benefits of the CRP program (Adler et al., 2009); however, much of this land is in areas of low precipitation, and yields could be below economic viability.

There are many types of marginal lands (Richards et al., 2014; Khanna et al., 2021) which can have different effects on the life cycle assessment (LCA) of biomass feedstock production. While drought prone sites have lower switchgrass yields, poorly drained sites may have similar yields to prime lands (Casler et al., 2017), resulting in lower inputs per unit of production; however, nitrous oxide (N_2_O) emissions may be greater on these poorly drained soils (Saha et al., 2017). Nitrogen (as nitrous oxide (N_2_O) and greenhouse gas (GHG) emissions associated with production of N fertilizers) and soil carbon (C) are the largest source and sink of GHG emissions, respectively, associated with feedstock productin (Adler et al., 2007; Adler et al., 2012). For switchgrass production, N_2_O emissions and GHG emissions associated with nitrogen (N) fertilizer production account for more than 80% of GHG emissions (Adler et al., 2012). With a lower requirement of N fertilizer (Maughan et al., 2012), Miscanthus could greatly reduce emissions (Saha et al., 2018) and water quality impacts (Rau et al., 2019) associated with feedstock production. On marginal lands with perennial grass vegetation, such as those in CRP, there is less potential for further sequestration of soil C (Gelfand et al., 2011). Prior vegetation, soil texture, and climate can all affect GHG emissions, leading to many options for designing landscapes to optimize GHG emissions (Field et al., 2018).

Understanding the variation in forage yields across the landscapes from field to subfield is important for crop placement in the landscapes to achieve both economic and environmental goals. Unfortunately, there is still a lack of publicly available yield data at landscape scale in the US for biomass crops such as Miscanthus. Most yield data are from plot scale plantings on research farms rather than marginal lands where commercial scale entities are planting the crop. Research plot yields are often much higher than commercial fields for a variety of reasons including reduced spatial variability [soils, establishment success] and location on better quality farmland (Wullschleger et al., 2010). Crop yields vary across the landscape with biophysical factors, such as climate, topographic attributes, and soils (Maestrini and Basso, 2018). These factors are very heterogeneous across the landscape, leading to large variations in crop yields, both within and between fields across the landscape. Modeling studies (Miguez et al., 2012; Song et al., 2015; Daly et al., 2018) and economic and life cycle assessments (Wang et al., 2012; Dwivedi et al., 2015; McCalmont et al., 2017a) have been based on these data, potentially leading to over-estimates of the yield potential of Miscanthus. Collection of high-resolution field scale yield data from commercial farms is important to understanding the spatial and temporal yield variability.

Satellite remote sensing has become an essential tool for measuring and monitoring terrestrial ecosystems over large areas because of its wide coverage, high spatial and temporal resolutions, and consistency (Yuan et al., 2007; Gu and Wylie, 2010). Satellite imagery has been used to estimate crop biomass yields (Johnson, 2014). In comparison to the commonly used Normalized Difference Vegetation Index (NDVI), the Enhanced Vegetation Index (EVI) has been shown to be more linearly correlated and less prone to saturation at high biomass yields (Jiang et al., 2008). Satellite imagery at moderate to high temporal and spatial resolutions can be used to track biomass production at broad spatial scales, and to identify areas in need of more intensive management intervention. Monitoring and analysis tools developed for biomass crops can be applied more widely to perennial and even annual crops.

Therefore, the objective of this study was to conduct life cycle inventory data for Miscanthus production based on actual commercial farm scale production inputs and yields. In addition, to evaluate the performance of the ASABE standard methods (ASABE, 2011) to characterize fuel use for agricultural farm machinery. We also evaluated the use of satellite imagery to characterize subfield Miscanthus cover and biomass yield.

## Materials and methods

### Site description

**Figure 1:**
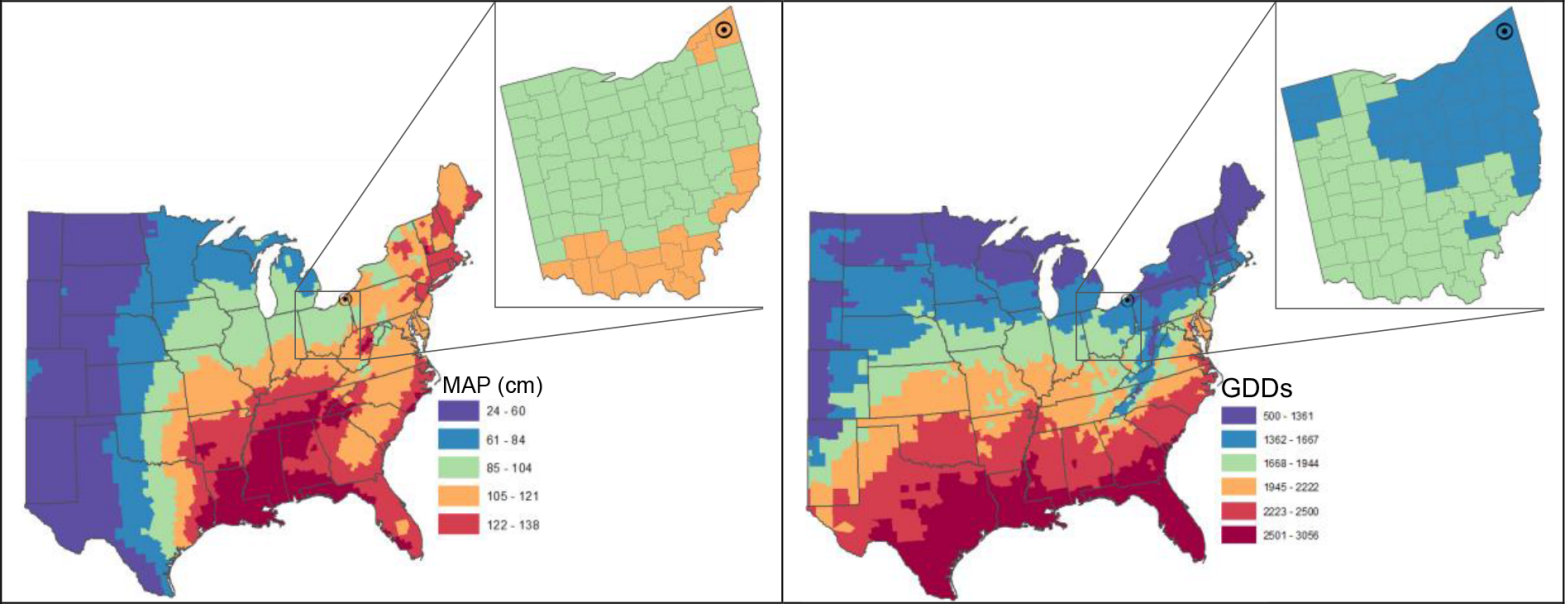
Mean annual precipitation [cm, 30 yr, 1981-2010] and growing degree days (base temperature, 10°C) [30 yr, April 15 to October 15, 1981-2010] of production region and the US.

Miscanthus production fields were located in Ashtabula County (Latitude 41.7, Longitude -80.7) in Northeastern Ohio (**Figure 1**) or parts of the surrounding 6 counties. The mean annual precipitation and growing degree days for this production region are shown in **Figure 1** with detailed monthly average temperature and total precipitation in **Table 1**. There were ~1,860 ha of Miscanthus under production. The soils in these production fields are a mixture [Mill silt loam (Fine-loamy, mixed, superactive, nonacid, mesic Aeric Epiaquepts), Platea (Fine-silty, mixed, active, mesic Aeric Fragiaqualfs), and Darien (Fine-loamy, mixed, active, mesic Aeric Endoaqualfs) silt loam]. They are very deep soils from somewhat to poorly drained.

**Table 1:** Monthly average of the mean daily temperature and total precipitation from 2012 to 2016 compared with the 30-yr average (1991-2020).

Month	2012	2013	2014	2015	2016	30-yr mean
Mean Air Temp, °C						
**Jan**	-1.7	-2.0	-8.1	-7.0	-4.1	-3.7
**Feb**	0.3	-3.9	-7.8	-12.6	-1.2	-3.0
**Mar**	8.8	-0.6	-3.3	-1.7	5.0	1.6
**Apr**	7.0	7.9	8.1	8.1	6.7	8.2
**May**	16.9	15.5	14.1	16.6	13.7	14.4
**Jun**	19.4	18.6	19.7	18.6	18.9	19.2
**Jul**	22.8	21.5	19.5	20.4	21.6	21.3
**Aug**	19.9	19.4	19.4	19.8	22.6	20.4
**Sep**	16.1	16.0	16.3	19.2	18.7	17.0
**Oct**	10.7	11.4	10.9	10.4	12.3	10.8
**Nov**	3.4	2.9	2.0	7.8	6.6	5.0
**Dec**	2.1	-1.5	1.2	5.6	-1.5	-0.4
Precipitation, mm						
**Jan**	107.6	59.3	67.4	96.3	45.9	88.8
**Feb**	48.6	51.7	68.9	61.5	94.1	61.0
**Mar**	88.5	54.1	59.7	45.2	87.4	80.1
**Apr**	38.9	84.8	119.4	89.4	86.7	102.0
**May**	85.6	90.1	106.1	155.3	88.0	96.6
**Jun**	68.1	245.1	172.6	193.5	59.2	111.4
**Jul**	69.1	199.7	146.3	68.1	121.8	114.0
**Aug**	82.9	118.1	128.0	72.5	142.2	95.9
**Sep**	123.3	92.4	84.8	122.8	146.2	107.8
**Oct**	202.7	139.2	121.6	95.4	128.6	109.4
**Nov**	59.0	124.0	82.7	70.4	51.4	94.1
**Dec**	141.4	99.8	64.8	113.6	135.1	94.2

### Miscanthus establishment

**Figure 2:**
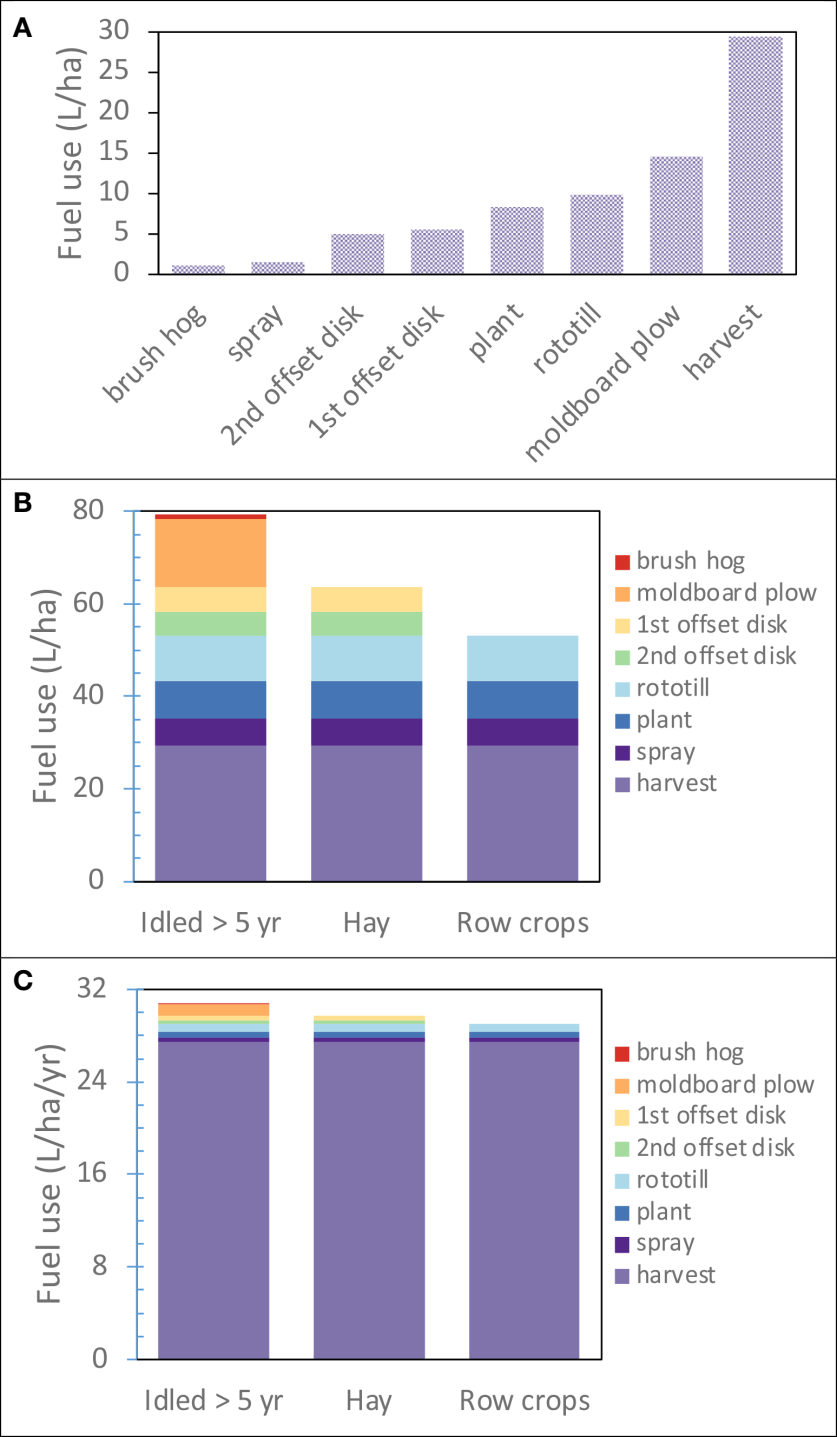
Fuel use for land preparation and Miscanthus production. **(A)** Fuel use for a single pass of the operation. Harvest includes both the forage harvester and forage wagon to collect chopped biomass directly discharged from the forage harvester in the field. **(B)** Cumulative fuel use for each operation over the three prior land use histories encountered: land idled for > 5 years, hayed, and row cropped. **(C)** Annual average fuel use over fifteen-year Miscanthus production cycle.

Prior land use history for the initial planting was about 15% row crop, 35% hay, and 50% idled for > 5 years. Establishment began in 2012 with ~90% established the first year and the remainder in 2013. Miscanthus (Miscanthus x giganteus (Mxg)) rhizomes were planted at a density of ~12,500 rhizomes/ha using a 4 row WHL planter (W.H. Loxton Ltd., UK; https://www.miscanthusplanter.com/) on 76.2 cm centers. Land preparation depended on prior land use history (**Figure 2**), with land idled for more than 5 years requiring brush hogging and moldboard plowing, and hayed land requiring 2 passes with an offset disk. Weed control consisted of a burndown prior to tillage using Roundup (4.48 kg ai/ha of glyphosate) and preemergence application of Degree Xtra (2.24 kg ai/ha of acetochlor and 1.12 kg ai/ha of atrazine) in each of the first three years either prior to planting Miscanthus in the first year or in each of the following two years prior to Miscanthus emergence in the spring. Although N fertilizer has shown a yield response in some regions (Maughan et al., 2012), a yield response was not measured in these fields, so no N was applied during production years.

### Farm data collection

**Figure 3:**
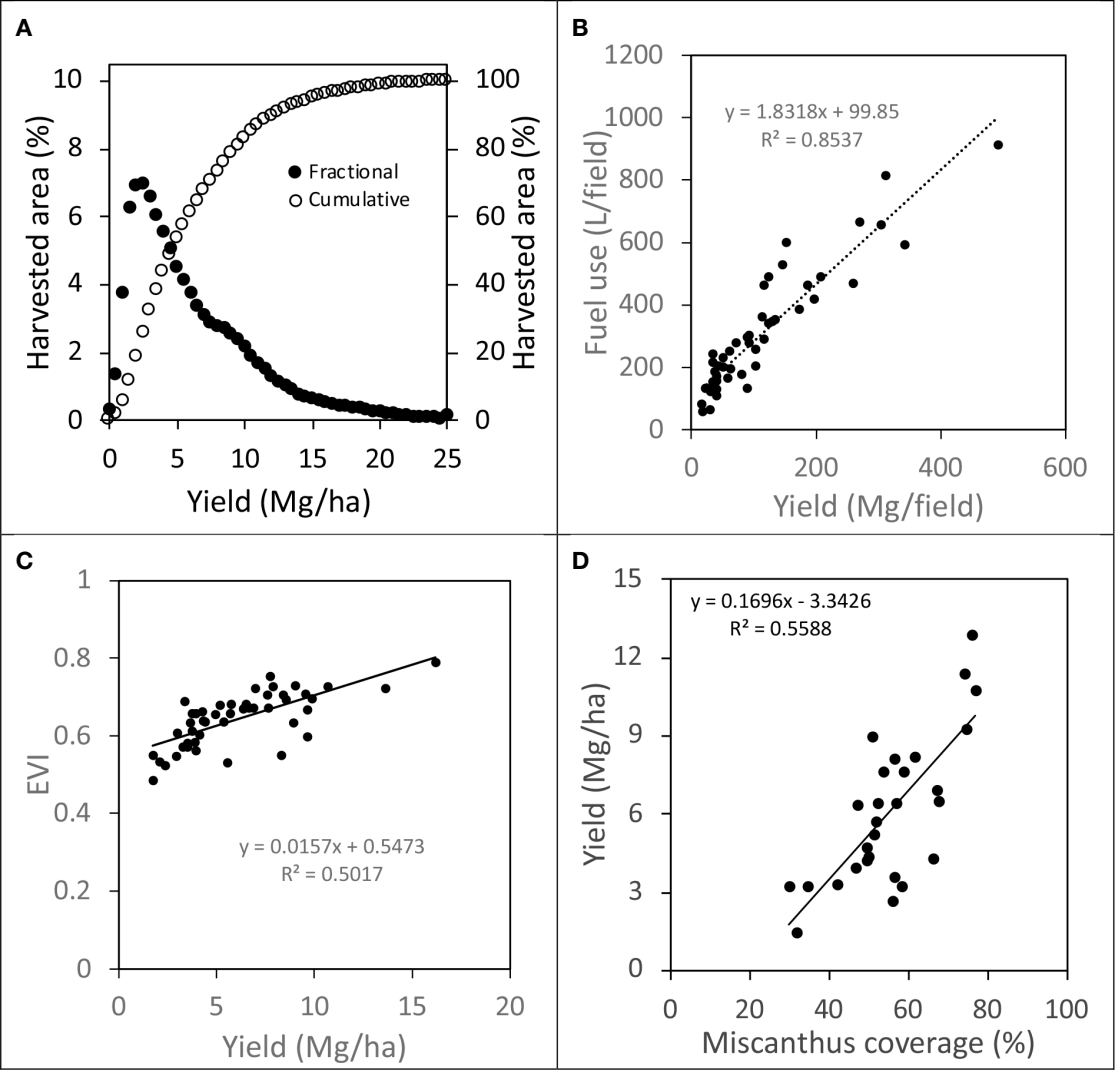
Characterization of Miscanthus yield within and across farm fields from the 2016 growing season: **(A)** subfield yield and harvested area [circles are the % harvested area for each 0.5 Mg/ha increment of yield across a total harvested area of ~855 ha], **(B)** total fuel use for harvest [Claas Jaguar 930 forage harvester only] and total biomass per field, **(C)** EVI from 30 m Landsat imagery and average field yield, and **(D)** field average yield and Miscanthus cover. Field sizes across the 48 different fields ranged from ~3.5 – 48.7 ha with a mean of ~17.8 ha and SD ~10.8 ha.

**Figure 4:**
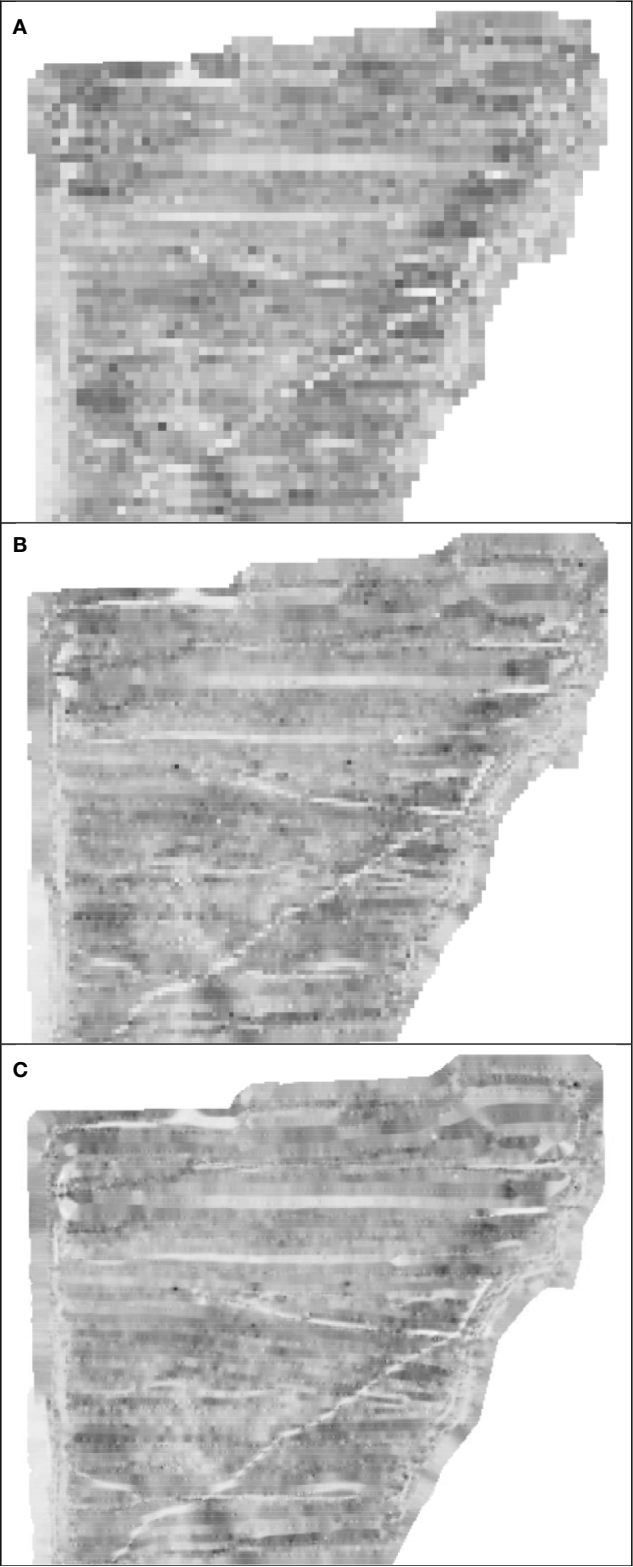
Spatial yield ranging from 0 to 22 Mg/ha across an example Miscanthus field with yield increasing with pixel darkness across a range of pixel resolutions **(A)** 10 m, **(B)** 5 m, and **(C)** 2 m.

We logged diesel fuel use on agricultural machinery for land preparation, establishment, and production of Miscanthus on ~855 ha of land using a J1939 Mini Logger™(HEM Data Corporation, Southfield, MI). The J1939 Mini Logger recorded fuel use as L/s and was georeferenced allowing identification of field and subfield location of the agricultural machinery and corresponding biomass yields. Miscanthus from the 2016 growing season was harvested at ~15 cm height after a killing frost from early winter to spring 2017 prior to regrowth using a Claas Jaguar 930 forage harvester. Chopped Miscanthus biomass was directly discharged into a Meyer RT200 Series forage box (Dorchester, WI) pulled by a Caterpillar Challenger 35 with tracks. Since the J1939 Mini Logger were not able to be installed on the Caterpillar Challenger 35, fuel use was calculated using the Nebraska Tractor Test Laboratory data (NTTL, 1995) assuming it was operated at 75% load and full throttle on average providing a fuel use of 35.9 l/h. Total fuel use for harvesting each field was quantified by summing all fuel use data while the harvester was within the field boundaries. To characterize spatial Miscanthus biomass yields, total field fuel use for harvest was indexed using total field biomass yield to generate an average value of L fuel/Mg biomass (**Figure 3**). When this index value was combined with harvester speed, a field yield map was generated (resolution ~12.5 m^2^/data point), and data extracted and binned into 0.5 Mg units and summed over area (**Figure 3**). To create an example spatial Miscanthus yield map (**Figure 4**), the Fishnet tool in ArcGIS Pro was used to create a 10 m grid. Since the chopping width of the forage harvester was ~9 m and data collected every second, there was an average of 8 data points per 10 m grid cell. The Inverse Distance Weighting (IDW) tool in ArcMap was used to interpolate a raster surface at 2, 5, and 10 m.

The three-band enhanced vegetation index [EVI] model described by Jiang et al. (2008) was generated from US Landsat 8 Analysis Ready Data (ARD) surface reflectance imagery from September 25 2016 at the time of peak Miscanthus biomass. The average EVI for all fields was regressed against the corresponding Miscanthus field average biomass yields (**Figure 3C**).

Google Earth imagery from September 2015 (15 cm resolution) was used to determine Miscanthus coverage in the fields. Coordinates from at least six, clearly defined features for each image were identified, recorded, and used to rectify the imagery in ArcGIS Desktop 10.5 using the Georeferencing tool (ESRI, 2023a). Once the imagery of all fields was rectified, an unsupervised classification of the RGB pixel values was performed using the Iso Cluster Unsupervised Classification tool (ESRI, 2023b). A total of 10 classes were used to group the pixels as either Miscanthus or non-Miscanthus and each class was manually assigned to a group. Miscanthus pixels were tabulated to determine the percent coverage in each field (**Figure 3D**).

### Life cycle assessment

Following ISO (2006) procedures, the life cycle assessment (LCA) of field operations was conducted to the edge of the field in two phases: 1) identify all sources of greenhouse gases for the life cycle inventory (LCI) of Miscanthus production and 2) quantify the impact of the sources of greenhouse gases on climate by converting the inventory to CO_2_ equivalents (life cycle impact assessment, LCIA).

### DayCent model simulations

To quantify changes in soil organic carbon (SOC) and soil N_2_O emissions during Miscanthus production for the LCA, we used the DayCent biogeochemical model (Del Grosso et al., 2012); see Adler et al. (2018) for more details about the model. DayCent has been shown to reliably represent plant growth and GHG fluxes for different biofuel crops including Miscanthus, showing a good relationship between observed and predicted carbon in both above and below ground (Davis et al., 2012; Hudiburg et al., 2015). Daily weather data for Ashtabula County Ohio required to drive DayCent were acquired from DAYMET (https://daymet.ornl.gov) and was selected from the 1-km cell that was closest to the area-weighted geographical center of cropped land. SSURGO (https://websoilsurvey.sc.egov.usda.gov) was used to characterize the soil texture from Miscanthus production fields.

Model outputs are sensitive to current SOC levels, which in turn are influenced by previous vegetation cover and land management. To acquire reasonable modern SOC levels, about 1788 years of native vegetation followed by plowing and about 220 years of cropping were simulated. A fire event and plow out of native forested land was assumed to occur in the year 1788. Historically accurate cropping systems were simulated, and improved cultivars, fertilizer applications, and tillage intensity were introduced at appropriate times. From 1789 to 1965, corn–wheat-grass hay rotations were common, no N fertilizer was applied, and conventional tillage was used. From 1966 to 1970, soybean was introduced and shifted cropping to a corn-soybean rotation with N fertilizer applied; in response to soil degradation, reduced tillage methods were adopted. From 1971 to 2010, three different land use history scenarios were modeled to align with lands planted to Miscanthus: corn-wheat-soybean rotation using fertilizers and no-tillage; continuous cool-season hay; and continuous cool-season hay fields abandoned in 2002 allowing shrubs and trees to re-establishing into the landscape. To transition from the prior land use scenario to establish Miscanthus, when following row crops, the establishment protocol included an application of a non-selective herbicide and strip rototilling. For continuous hay, the same management was used as row crops with the addition of two offset disking events just prior to rototilling. In the final scenario for abandoned hay fields, the fields were mowed, and moldboard plowed in addition to the herbicide, disking, and rototilling.

**Figure 5:**
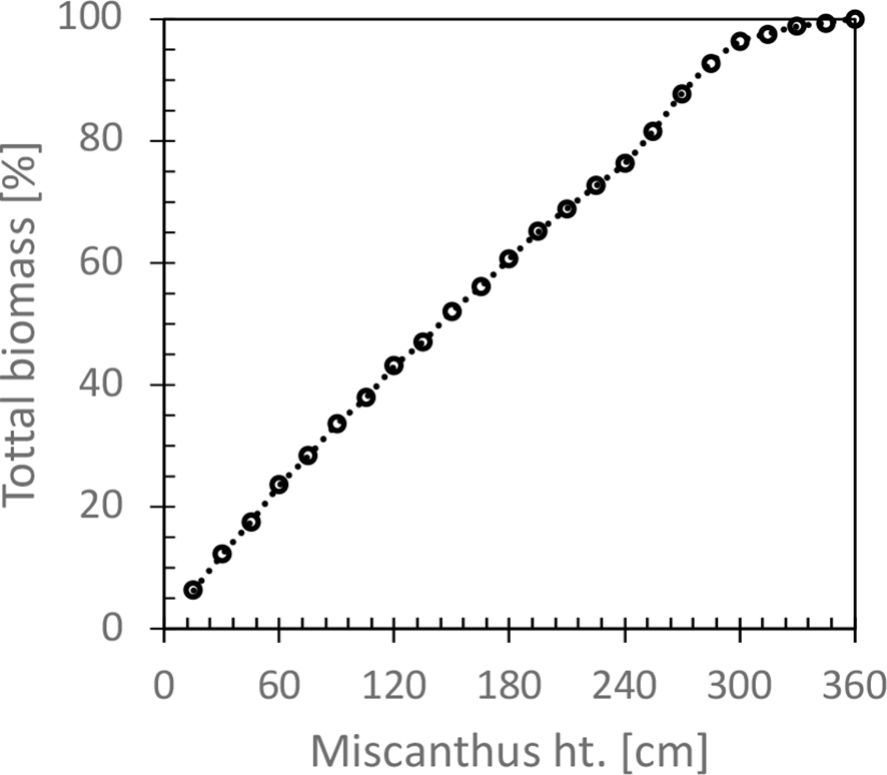
Relationship between Miscanthus height and percent of total biomass. Shoots were harvested in October prior to leaf drop. Segments included stems (~67% of total biomass), leaves (~29%), and panicles (~4%).

Simulations of changes in soil N_2_O emissions and SOC fluxes using DayCent were run for 15 years following establishment of Miscanthus, assuming a prior land use history of hay lands for 40 years. Unharvested Miscanthus residue was ~30% (**Figure 5**). See Magenau et al. (2021) for detailed results on harvest height and biomass yield. Soil N_2_O emissions were calculated as the sum of direct and indirect N_2_O. The direct N_2_O was the mean annual N_2_O emissions over the simulation period. To calculate indirect N_2_O, we combined DayCent outputs for NO_3_ leached and N volatilized with IPCC (de Klein et al., 2006) methodology. IPCC (de Klein et al., 2006) methodology assumes that 0.75% of NO_3_-N leached is eventually denitrified to N_2_O-N in water ways and that 1% of volatilized N (NO_x_+NH_3_) is deposited on soil and converted to N_2_O. N_2_O emissions were converted to CO_2_e by assuming that its global warming potential is 298 times that of CO_2_ on a mass basis (Forster et al., 2007). The GWI of diesel (92 g CO_2_e MJ^-1^) as documented by US EPA (2010) and includes end use in a light-duty internal combustion vehicle. The CO_2_e emissions associated with the manufacture of chemical farm inputs (i.e. herbicides) were from West and Marland (2002).

## Results

Diesel fuel use for agricultural machinery of individual operations used for land preparation and Miscanthus establishment and production ranged from 1 to almost 30 L/ha (**Figure 2A**). Brush hog and moldboard plow were only used on land idled for more than 5 years, while offset disks were also used on hay land. The highest fuel use for land preparation and Miscanthus establishment was on land idled for more than 5 years, ~50 L/ha (**Figure 2B**), while for hay land it was ~34 L/ha, and when row crops were the prior land use, little tillage was required leading to a fuel use of ~24 L/ha. Although there were significant differences in fuel use for land preparation, when fuel use was annualized over a 15-year time frame, biomass harvest accounted for ~90-95% (**Figure 2C**).

Collection of fuel use for establishment and management of commercial Miscanthus fields demonstrated that the use of ASAE standard methods was appropriate to quantify fuel use for crop production. When field measurements of fuel use were compared with those calculated using the ASABE standard methods (ASABE, 2011), they were all within 20% (**Table 2**). Field measurement of the second pass of the offset disks and moldboard plow were only one and two percent higher, respectively, than the ASABE standard calculations (**Table 2**). Field measurement of the forage harvester and first pass of the offset disk were 12 and 17% lower than the ASABE standard calculations, while the moldboard plow and second pass of the offset disk were within 2%.

**Table 2:** Comparison of field measurements of diesel fuel use for land preparation and Miscanthus establishment and production compared with ASABE standard methods (ASABE, 2011).

	Fuel	Time	Fuel	ASABE
	efficiency	efficiency	rate	standard
Implement	[L/ha]	[ha/h]	[L/h]	(L/h)
Moldboard plow	14.6	1.7	24.6	24.1
Offset disk 1st pass	5.6	3.6	19.8	23.8
Offset disk 2nd pass	5.0	4.2	20.7	20.4
Forage harvester direct cut	18.7	3.3	60.3	68.8

Miscanthus biomass yields were variable within the fields, as captured by both fuel use for biomass harvest and EVI. Characterizing the within field yield variability, ~1% of land had yields greater than 20 Mg/ha, ~18% had yield between 10-20 Mg/ha, and ~32% yields were between 5-10 Mg/ha (**Figure 3A**), with the remainder (~49%) < 5 Mg/ha, three to four years after establishment. Although Miscanthus biomass yield was variable both between and within fields, there was a good relationship between measured field yields and fuel to harvest fields (R^2^ = 0.85) (**Figure 3B**) and between measured field yields and EVI (R^2^ = 0.50) (**Figure 3C**). Much of the within field biomass yield variability could be explained by variation in Miscanthus cover (R^2^ = 0.56) (**Figure 3D**). An example of the spatial variability of Miscanthus yield can be seen in **Figure 4**, where yields varied from 0 to 22 Mg/ha over a range of data resolutions (2, 5, 10 m).

**Figure 6:**
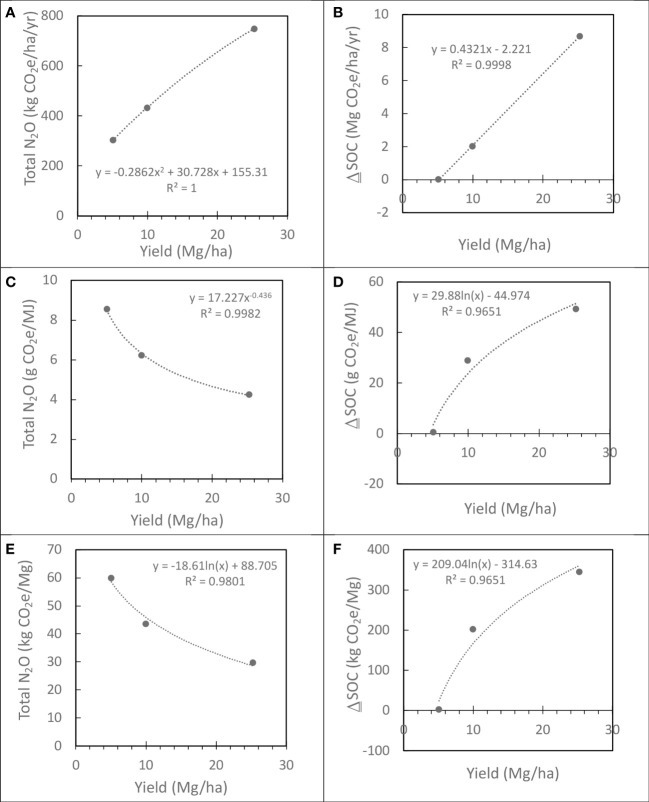
Variability in soil N_2_O emissions and changes in soil carbon on a unit area, fuel, and biomass basis averaged across the 15-year simulation period. Values [see **Table 3**] are area weighted for prior land use history [15% row crop, 35% hay, and 50% idled for > 5 years].

**Figure 7:**
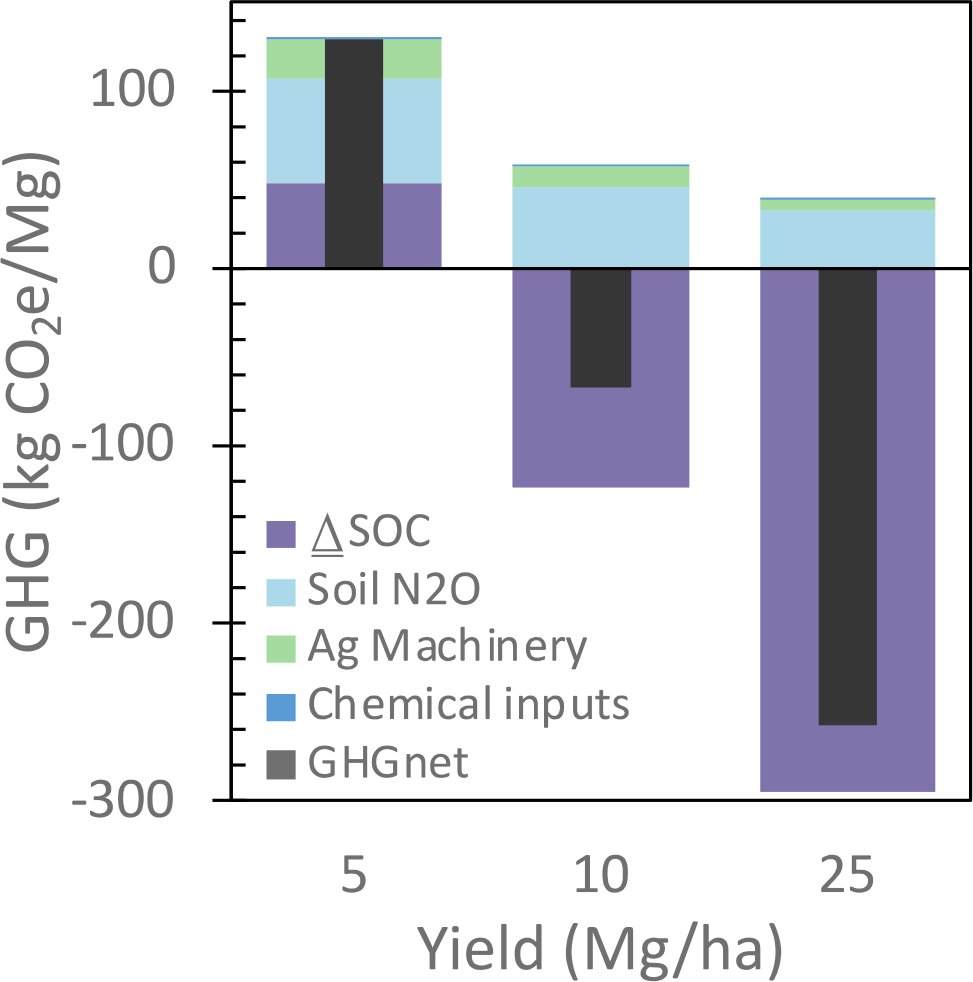
Life cycle greenhouse gas emissions over a range of Miscanthus yields during a fifteen-year production cycle are area weighted for prior land use history [see **Figure 6**].

Both N_2_O emissions and ΔSOC varied with prior land use history and Miscanthus yield and whether change was expressed on a unit area or yield basis (**Table 3**). N_2_O emissions decreased with prior land use history from row crop > hay > idle lands whether expressed on a unit area or yield bases and increase with yield on a unit area basis and decrease on a unit yield basis (**Table 3**). ΔSOC increased with prior land use history from row crop < hay < idle lands whether expressed on a unit area or yield bases and increase with yield on a unit area basis and yield basis. When prior land use N_2_O emissions and ΔSOC values were weighted by land area represented by each prior land use history, the trends were the same as the individual component prior land use practices. N_2_O emissions and ΔSOC increased with Miscanthus yield on a unit area basis (**Figure 6A, B**). However, on a MJ ethanol basis (**Figure 6C, D**) or Mg biomass basis (**Figure 6E, F**), N_2_O emissions decreased and ΔSOC increased with Miscanthus yield. Over the 15-year simulation period, Miscanthus yield needed to be >5 Mg/ha for ΔSOC to be positive. Since N_2_O emissions and ΔSOC were the largest source and sink for greenhouse gas emissions for Miscanthus production, respectively, and varied with yield, the net GHG varied with yield being positive for yields < 5 Mg/ha and negative yields > 10 Mg/ha (**Figure 7**).

**Table 3:** Comparison of prior land use history on soil N_2_O emissions and change in soil carbon over a 15-year time period from establishment using DayCent.

Prior	Greenhouse gas		Biomass yield (Mg/ha/yr)		
land use	source/sink	Units	5	10	25
Row crop	Total N_2_O	(kg CO_2_e/ha/yr)	325	449	766
		(kg CO_2_e/Mg)	64.1	45.2	30.4
		(g CO_2_e/MJ)	9.17	6.46	4.34
	SOC	(Mg CO_2_e/ha/yr)	-0.83	1.16	7.83
		(kg CO_2_e/Mg)	-164	117	311
		(g CO_2_e/MJ)	-23.5	16.7	44.4
Hay	Total N_2_O	(kg CO_2_e/ha/yr)	306	435	754
		(kg CO_2_e/Mg)	60.3	43.8	29.9
		(g CO_2_e/MJ)	8.62	6.25	4.27
	ΔSOC	(Mg CO_2_e/ha/yr)	-0.31	1.69	8.37
		(kg CO_2_e/Mg)	-61	170	332
		(g CO_2_e/MJ)	-8.7	24.3	47.4
Idle > 5 years	Total N_2_O	(kg CO_2_e/ha/yr)	296	426	740
		(kg CO_2_e/Mg)	58.4	42.8	29.3
		(g CO_2_e/MJ)	8.35	6.12	4.19
	ΔSOC	(Mg CO_2_e/ha/yr)	0.50	2.50	9.18
		(kg CO_2_e/Mg)	98	251	364
		(g CO_2_e/MJ)	14.0	35.9	52.0

## Discussion

Consistent with other studies of various cropping systems modeling biogeochemical changes in soil carbon and nitrogen with crop production (Adler et al., 2007; Wang et al., 2012; Adler et al., 2018), soil N_2_O emissions were the largest source, and soil carbon sequestration the largest sink of greenhouse gas emissions during Miscanthus production. Modeled soil N_2_O emissions and changes in soil carbon were similar with previous field measurements. The predicted N_2_O values were in the range observed in field studies (Holder et al., 2019a; Zeri et al., 2020). In addition to the soil environment affecting N_2_O emission in Miscanthus fields (Saha et al., 2017; Saha et al., 2018), yield may also be a contributing factor (**Figure 6**). Yield will vary spatially due to variation in biophysical factors across and within fields and from this study, we would expect soil carbon to increase with biomass yields. It took a relatively long time for soil carbon to gain back loses during land preparation for Miscanthus production as previously observed in Holder et al. (2019b). In this study, ~49% of the harvested area with < 5 Mg/ha biomass may not be sequestering further SOC (**Figure 6B**). The changes in SOC predicted in this study were similar to other studies (Clifton-Brown et al., 2007; Agostini et al., 2015; McCalmont et al., 2017b; Richter et al., 2015; Robertson et al., 2017). Within similar climates, we expect soil carbon to vary with biomass yield, time from establishment, and prior land use.

Although fuel use was higher for land preparation in fields on land idled for >5 years or hay lands, than fields with row crops, greenhouse gas emissions from agricultural machinery were small compared with soil N_2_O emissions and ΔSOC. We have shown this previously in studies with different cropping systems (Adler et al., 2007; Adler et al., 2018). Although land preparation including Miscanthus establishment consumed more energy than a single harvest event, since harvest is an annual event, over a 15-year time frame, it dominated energy use for Miscanthus production. Fuel to harvest Miscanthus dominated greenhouse gas (GHG) emissions from agriculture machinery for Miscanthus establishment and management. This could be different with annual cropping systems where land preparation occurs annually in contrast with perennial cropping systems. This study confirms that using the ASABE standard methods for estimating diesel fuel use for various farm operations did a good job estimating field scale fuel use.

For new crops such as Miscanthus where commercial scale production fields are rare, small plot trials are have been viewed as the best available data to base and evaluate predictive models and consequently what they are based on (Miguez et al., 2012; Song et al., 2015; Daly et al., 2018). In contrast, this study measured Miscanthus biomass yields on a commercial farm scale. Modeling studies for Miscanthus over the US (Miguez et al., 2012; Song et al., 2015; Daly et al., 2018) overestimated the biomass yields in the region in this study by two to three times. Miscanthus yields on commercial production fields have been previously quantified in the UK where on-farm yields averaged ~9 Mg/ha (Richter et al., 2016), more similar to yields measured in this study. In this study, the average yield across ~855 ha was ~6.2 Mg/ha, 3-4 years after establishment.

Understanding the reasons for spatial variability in Miscanthus yield can help better target the location of production fields. That wasn’t the focus of this study, but we did observe that Miscanthus cover was related to biomass yield. Although it wasn’t clear what caused the poor establishment and spatial yield variation, these soils are poorly drained and it was thought that was probably the main limiting factor for productivity. Precipitation during the 2012 establishment year was low, about 60% of the 30-year average for June and July, progressively making up the deficit over the summer; the annual precipitation was similar to the long term 30-year average (**Table 1**). Richter et al. (2016) also found that soil moisture was a limiting factor at their site. Although poor establishment could account for the cover and low yields, gaps are difficult to repair at commercial scale. Some soil areas were clearly higher yielding, some areas of poor yield were missing plants and may have been due to drought during establishment. However other areas had stunted plants, which is consistent with a constraining soil environment.

Satellite imagery has potential to capture spatial Miscanthus biomass yield and better understand yields across biophysical gradients using field scale yield data. We showed that there was a good relationship between EVI from Landsat imagery and field scale Miscanthus biomass yields, supporting the potential application of using satellite imagery to predict Miscanthus biomass yields in other regions of the US. Richter et al. (2016) made a similar observation using NDVI that it did a good job capturing field scale yields. There was also a good relationship between fuel use for Miscanthus biomass harvest and field scale biomass yield. Using fuel use for Miscanthus biomass harvest as a proxy for within field biomass yields, has the potential for better understanding subfield spatial yield variability due to biophysical factors such as topography and soils. Soil properties and topography show promise to explain spatial biomass yield variability. In this region, poorly drained soils seem to drive much of the biomass yield variability.

## Data availability statement

The original contributions presented in the study are included in the article, further inquiries can be directed to the corresponding author.

## Author contributions

The author confirms being the sole contributor of this work and has approved it for publication.
